# Lactobacillomics as a new notion in lactic acid bacteria research through omics integration

**DOI:** 10.1007/s11274-025-04285-y

**Published:** 2025-02-11

**Authors:** Özge Kahraman Ilıkkan

**Affiliations:** Kahramankazan Vocational School, Department of Food Processing, Başkent University, Ankara, Türkiye Turkey

**Keywords:** Lactobacillomics, Omics, Lactic acid bacteria, Probiotics, LAB

## Abstract

Omics technologies are a set of disciplines that analyze large-scale molecular data to understand biological systems in a holistic way. These technologies aim to reveal the structure, functions and interactions of organisms by studying processes at many levels of biomolecules, from the genome to metabolism. Lactobacillomics is introduced as an interdisciplinary field that integrates multiple “omics” technologies—including genomics, transcriptomics, proteomics, metabolomics, and metagenomics— to provide a comprehensive insight into “lactic acid bacteria” species. Lactobacillomics aims to elucidate the genetic, metabolic, and functional characteristics of lactic acid bacteria (LAB) species, providing insights into the mechanisms underlying their probiotic effects and contributions to the host microbiome. By analyzing genomes and metabolic pathways, researchers can identify specific genes responsible for health-promoting functions and desirable fermentation characteristics, which can guide the development of targeted probiotic strains with optimized health benefits. The integration of these omics data allows facilitating the discovery of biomarkers for health and disease states, the development of new probiotics tailored to specific populations or health conditions, and the optimization of fermentation processes to enhance the safety, flavor, and nutritional profile of fermented foods. A comprehensive review and bibliometric analysis were conducted to provide an overview of this promising field between 2005 and 2025 by examining Web of Science Core Collection data. Research results reveal trending topics, future perspectives, and key areas of growth within lactic acid bacteria (LAB) studies, particularly as they intersect with omics technologies.

## Introduction

Lactobacillomics is a comprehensive approach to the study of lactic acid bacteria (LAB) species and related probiotics by using omics technologies. As a framework, Lactobacillomics encompasses various disciplines of biological information, including genomics, transcriptomics, proteomics, metabolomics, and other omics methodologies, to deeply understand the functional and ecological characteristics of LAB, which includes six families (*Aerococcaceae*,* Carnobacteriaceae*,* Enterococcaceae*,* Lactobacillaceae*,* Leuconostocaceae* and *Streptococcaceae*), 33 genera, and about 300 species (Zheng et al. 2020; Qiao et al. 2022; Rossi 2023; Walter and O’toole 2023). Especially, *Lactobacillus* spp. and other related genera according to recent classification such as *Lactiplantibacillus*,* Ligilactobacillus*, *Limosilactobacillus*,* Lentilactobacillus*, and *Levilactobacillus* (Zheng et al. 2020).

Omics technologies are at the forefront of modern biotechnology and microbiology due to their ability to systematically analyze complex biological data. Fundamental omics techniques relevant to Lactobacillomics include metagenomics, metataxonomics, metabonomics, microbiomics, proteomics, transcriptomics, lipidomics, and metabolomics (Morrison et al. 2006; Capozzi and Bordoni 2013; Patrinos et al. 2020). The main applications of these approaches can be defined as follows; genomics, profiling DNA, transcriptomics, measuring transcripts, proteomics and metabolomics, quantifying proteins and metabolites. Omics technologies are among the most recent and innovative approaches in the fields of biotechnology and microbiology (Capozzi and Bordoni 2013; Stefanovic et al. 2017). The study of LAB using omics technologies could lead to discoveries in this area. So, combining these elements, “Lactobacillomics” could refer to a comprehensive study of LAB. This might involve understanding their genetic makeup (genomics), the proteins they produce (proteomics), the metabolites they produce (metabolomics), and their interactions within a host organism or their ecological role in various environments. Genomics and metagenomics enable the identification of genes responsible for probiotic traits, metabolic pathways, and resistance mechanisms as well as proving a complete view of microbial communities containing LAB such as the human gut or fermented food ecosystems. This approach helps identify LAB’s role in microbial interactions and functional contributions in diverse environments. Transcriptomics provides a dynamic perspective, uncovering gene expression patterns under various environmental conditions, which is essential for understanding LAB adaptation and functionality. Proteomics delves into the proteome, revealing the functional proteins involved in metabolic processes, stress response, and host interactions. This is crucial for linking genotype to phenotype in LAB studies. Metabolomics focuses on the small molecules produced by LAB, offering insights into their metabolic outputs, such as organic acids and bioactive compounds, which are critical for their probiotic effects and industrial applications.

The term “lactic acid bacteria” (LAB) refers to a class of Gram-positive bacteria that generates lactic acid through the metabolism of carbohydrates and the genus *Lactobacillus* includes over 260 species (Salvetti et al. 2018; Gu and Zhao 2019; Walter and O’toole 2023). The utilization of LAB has been dated very long ago in history. Even though fermented foods are the main sources of LAB, these bacteria have different application areas such as food production and processing, and medical and pharmaceutical applications. In addition, polysaccharides called exopolysaccharides (EPS) produced by LAB also have a wide range of applications. Understanding these bacteria’s potential more thoroughly through omics technologies could contribute to the development of applications in these fields (Stefanovic et al. 2017). EPS has been shown to have wound healing effects on colonic cells, immunoregulatory potential, anticancer activity, periodontal regeneration potential (Ciszek-Lenda et al. 2011; El Ghany et al. 2015; Kibar et al. 2020; Wu et al. 2021; Kahraman-Ilıkkan et al. 2024).

Examples of omics studies conducted with LAB are vitamins, volatile compounds, and amino acid analysis through metabolomics, characterization with high-throughput sequencing, and environmental stress responses of LAB such as heat shock, cold shock, and acid shock through transcriptomics and proteomics.

LAB has been revealed to be rich for the tryptophan metabolism genes by multi-omics approach. LAB exhibited a strain specific production of indole-3-lactic acid (ILA), indole-3-acetic acid, and 3-indolealdehyde (IAld). *Ligilactobacillus salivarius* has been shown to produce indole-3-lactic acid (ILA) (Pan et al. 2023a). Another potential application of LAB is in psychobiotics. *Lactobacillus reuteri* relieved depressive-like behavior by providing serotonin synthesis. *Lactobacillus reuteri* was found to have an anti-depressive effect by improving the intestinal microbiota determined by metagenomic method (Xie et al. 2020). Key pathways and metabolites can be identified by using multi-omics techniques. *L. plantarum* has anti-obesity potential by producing some metabolites such as N6 N6-Dimethyl‐l‐Lysine, which is detected by metabolomics (Su et al. 2024).

## Methodology and data collection

Bibliometric analysis is a quantitative method used to assess the impact, development, and trends of published academic literature. The bibliometric analysis provides insights into the interconnectedness of research topics, institutions, countries, or individual researchers by examining various metrics associated with publications (Aria and Cuccurullo 2017; Wang et al. 2023a). In this study, relevant research papers and book chapters published in English were collected, examined, and summarized to capture key insights. The analysis was conducted using VOSviewer version 1.6.20 and RStudio version 2024.09.0. The PRISMA 2020 Flow Diagram was generated with the help of the web page (https://estech.shinyapps.io/prisma_flowdiagram/) (Haddaway et al. 2022). RStudio bibliometrix library and Biblioshiny were used to visualize data (Aria and Cuccurullo 2017). The data collection occurred in October 2024, focusing on a comprehensive literature review and bibliometric analysis spanning the years 2005 to 2025. This timeframe was chosen to allow for an in-depth examination of the development of omics technologies and advancements in research on LAB, as fields such as genomics and proteomics have experienced significant growth since 2005. Science Citation Index Expanded, Emerging Sources Citation Index, and Book Citation Index of the Web of Science Core Collection (WoSCC) were selected. The search terms were selected as follows; “omic* technologies” OR “genomic*” " OR “proteomic*” OR “transcriptomic*” OR “metabolomic*” OR “functional genomic*” OR “microbial metabolism” OR “multi-omic*” OR “Metabonomic*” OR “metagenom*” OR “metataxonomic*” OR “metabolic engineering” OR “foodomic*” OR “lipidomic*” AND “lactic acid bacteria”. An asterisk (*) was used to include all variations of a term, ensuring comprehensive data capture (e.g., “genomics” or “genomic”).

### Applications of omics technologies to LAB

These sections are a solid starting point for covering the applications of omics technologies to LAB within the context of Lactobacillomics. Each subsection focuses on a core “omics” approach that provides critical insights into LAB species.

### Genomics in lactobacillomics approach

The key points in this section include an overview of genome sequencing methods and their application to LAB, identifying key genetic markers in LAB species that contribute to probiotic functions, and using comparative genomics to understand species diversity and functional genes across strains. Research provides a comprehensive overview of how genomic approaches are applied to understand LAB species’ diversity, adaptation, and probiotic potential within the scope of Lactobacillomics. Recent research in the genomics of LAB highlights the versatility and applications of these bacteria, especially in fields like food production, probiotics, and biotechnology. Advances in genome sequencing and gene editing are revealing LAB’s genetic adaptations to various ecological niches, including fermented foods and the human gut. This adaptation process involves both genome reduction in certain strains and the acquisition of niche-specific genes, often found on plasmids or near prophages. This combination of reductive evolution (losing genes or genetic material over time) and gene acquisition (a process by which an organism gains new genes, typically through horizontal gene transfer (HGT) supports LAB’s diverse functionality in nutrient processing and environmental adaptation. LAB research also focuses on enhancing beneficial traits through genome editing. For example, genome modifications are being applied to optimize LAB for enhanced probiotic efficacy, nutrient synthesis, and pathogen resistance; while ensuring they remain safe for consumption. Genetic tools now enable targeted alterations in LAB genomes, advancing strain development for specific applications such as bio-enriched foods and antimicrobial compounds. These developments have potential applications in industrial fermentation, biotherapeutics, and even the production of certain vitamins (Börner et al. 2019a).

Genome analysis using next-generation sequencing (a high-throughput DNA sequencing technologies) has advanced the characterization of LAB, providing insights into their adaptation in food fermentations and interactions with the human host (Douillard and de Vos 2014). Recently, whole genome sequencing has been utilized to identify the genomes of lactobacilli (Kim et al. 2022; Kahraman-Ilıkkan 2024). This method not only facilitates identification but also provides an overview of the safety or risk assessment of strains by analyzing virulence factors, resistomes, and biogenic amine production (Peng et al. 2023). Additionally, genes associated with probiotic bacteria such as *strA* (cell wall adherence), *bsh* (bile salt hydrolase), *chol* (Choloylglycine hydrolase), *dnaK/J* (chaperons) can be identified through genome analysis (Kahraman-Ilıkkan 2024). Furthermore, CRISPR/Cas systems can be examined by analyzing these genomes. This analysis is important for utilizing the bacteria’s own CRISPR/Cas systems for gene editing (Kahraman Ilıkkan 2021a, b).

Genetic adaptations linked to thermal tolerance have been detailed in the genome sequencing of a *L. acidophilus* strain engineered for enhanced heat resistance (Jeon et al. 2021). Another study examined *Limosilactobacillus fermentum* LAB-1, a lactic acid bacterium, highlighting its robust metabolic and probiotic capabilities. Genomic analysis uncovered genes supporting carbohydrate and amino acid metabolism, and adaptation systems like CRISPR-Cas for phage defense. LAB-1 synthesizes B-group and K vitamins, essential for enriched foods, and shows potential for antimicrobial production. Additionally, it has desirable probiotic traits, including flavor and exopolysaccharide production, without harmful antibiotic resistance, indicating its safety and utility for food, biotechnology, and health applications (Hossain 2022).

Future research directions and potential applications of genomic studies in LAB in the Lactobacillomics approach are generally included in LAB’s therapeutic potential, such as developing probiotics that target specific diseases, gastrointestinal disorders, obesity, and metabolic syndrome. With the rise of functional foods, genomic tools can be utilized to enhance the production of bioactive compounds from LAB in fermented products. This includes optimizing metabolic pathways for higher yields of beneficial metabolites like short-chain fatty acids and vitamins and improving the health benefits of fermented foods (Carvalho and Conte-Junior 2024). Future research may also focus on utilizing LAB in sustainable food systems, including their role in waste valorization processes, where LAB can help in the solid-state fermentation of food waste into value-added products. Genomic studies can identify metabolic pathways that improve LAB efficiency in these processes, contributing to sustainability efforts in food production (Rachwał and Gustaw 2024). Genomic tools can help elucidate how specific LAB strains interact with the immune system and gut microbiota, providing insights into their mechanisms of action and potential therapeutic effects (Karthika Parvathy et al. 2022).

### Transcriptomics in lactobacillomics approach

Transcriptomics allows researchers to map out the active genes in LAB strains under specific conditions, such as during gut colonization, dairy fermentation, or exposure to different pH levels (Gu and Zhao 2019). By examining the transcriptomic responses of LAB strains to heat, oxidative, or acid stress, researchers can understand survival mechanisms. For example, insights into heat shock proteins or acid tolerance genes aid in identifying strains that are more resilient in challenging environments, like the gastrointestinal tract. When combined with genomics, proteomics, and metabolomics, transcriptomics provides a more comprehensive view of LAB species, helping researchers understand not only which genes are expressed but also how these expressions translate into metabolic functions and products. This holistic approach is invaluable for optimizing strains for specific industrial or therapeutic purposes.

Pre-adapting *Lactiplantibacillus pentosus* strains to edible oils is an innovative strategy aimed at enhancing their robustness, particularly in the context of antibiotic resistance. By exposing these probiotic strains to different vegetable-based oils like olive, sunflower, argan, and linseed oils researchers examined how this pre-adaptation could alter both phenotypic and genotypic responses to antibiotics. The study identified that oil pre-adaptation influenced the expression of genes such as *rps*L, *rec*A, and *uvr*B, which are associated with stress responses. This highlights how environmental exposures, like those in the food matrix, may induce protective or adaptive gene expressions. Specifically, *recA* is known for its role in DNA repair and genetic recombination, which can play a part in resistance development, while *rps*L and *uvr*B are involved in ribosomal function and DNA repair under stress (Alonso Garcia et al. 2023). These findings have practical implications for creating more stable and robust probiotic products. By pre-adapting *L. pentosus* strains to oils like olive or sunflower, it may be possible to produce probiotic strains that not only exhibit enhanced survival under gut conditions but also possess functional benefits that enhance their safety and effectiveness. This approach could support probiotic resilience in food matrices rich in fats or oils, such as dairy or plant-based products, potentially expanding the scope and functionality of probiotics in different diets.

The study of the preventive potential of probiotics, specifically *Bifidobacterium bifidum* FL-228.1, in protecting the intestinal barrier highlights the growing interest in using probiotics to support gut health. Unlike many studies focusing on probiotics as a remedy for pre-existing gut issues, this research examines whether certain strains can act prophylactically to fortify the intestinal barrier, thus providing resilience against potential harm. The tested strains modulated immune responses by adjusting the ratio of interleukins, specifically IL-10 (anti-inflammatory) and IL-12 (pro-inflammatory) in peripheral blood mononuclear cells (PBMCs). This modulation can promote an anti-inflammatory state, which is beneficial for maintaining a healthy intestinal barrier. Transcriptomic analysis and protein-protein interaction (PPI) studies pointed to several pathways through which FL-228.1 may exert its effects as NLRP3 inhibition, PPARγ activation, and TLR2 activation (Wang et al. 2023b).

Another study investigated the nutritional potential of *Lactiplantibacillus plantarum* A6 in a food matrix using next-generation sequencing techniques. By analyzing both the genome and transcriptome of *L. plantarum* A6, the researchers aimed to uncover its unique nutritional contributions when cultivated in a pearl millet food matrix compared to a laboratory medium. Transcriptomic analysis identified significant differences in gene expression depending on the cultivation environment. The genome of *L. plantarum* A6 was compared with several other *L. plantarum* strains (WCFS1, ST-III, JDM1, and ATCC14917), revealing five regions of genomic plasticity. These regions contained 362 coding sequences specific to *L. plantarum* A6, many of which code for proteins with unknown functions. This genomic uniqueness has been thought to contribute to the strain’s distinct metabolic capabilities (Turpin et al. 2018).

Another study examined the protective effects of green tea polyphenols (GTP) on *Enterococcus faecalis* (a lactic acid bacterium commonly used as a probiotic) when exposed to bile salt stress, a challenging condition often encountered in the gastrointestinal tract. By using RNA sequencing, the researchers analyzed how GTP influences gene expression in *E. faecalis*, with a focus on mechanisms that support cell integrity, nutrient transport, and metabolic stability under stress (Zhang et al. 2022).

Heat tolerance is crucial for *L. plantarum* to survive manufacturing processes, such as pasteurization, that involve high temperatures. The genetic and molecular mechanisms that contribute to heat resistance in *Lactiplantibacillus plantarum* CGMCC8198, a probiotic bacterium widely used in functional foods, have been extensively studied. Acclimation, a process of gradually exposing bacteria to sublethal heat stress, was investigated to boost the heat resistance of *L. plantarum*. Through transcriptomic and bioinformatics analysis, four key genes—*adh*E-like (alcohol dehydrogenase), *his*E (phosphoribose-ATP pyrophosphatase), *yku*N (flavodoxin), and *fol*B (dihydropterin aldolase)—were identified as potentially linked to heat resistance in *L. plantarum*. These genes are associated with metabolic activities, antioxidant defense, and cell wall stability, which are essential for bacterial survival under stress. The findings indicate that *adh*E-like, *his*E, *yku*N, and *fol*B play significant roles in the heat resistance of *L. plantarum*, highlighting their potential as genetic markers for selecting or engineering heat-resistant probiotic strains. These genes could be further explored to create strains that are more robust during manufacturing and storage, ensuring higher survival rates and effectiveness in functional foods (Da et al. 2023).

Transcriptome was also used for characterization. Using a combination of anaerobic culturing, culturomics, and metagenomic analysis, 305 LAB strains were isolated from canine feces. Four strains; *Lactobacillus amylolyticus*,* Ligilactobacillus salivarius*,* Enterococcus hirae*, and *Enterococcus faecium* were identified as promising probiotics. These strains stood out because of their ability to extend the lifespan of *C. elegans* and mitigate aging-related neuronal degeneration. Through whole transcriptome analysis and integrative network analysis, the researchers observed changes in various mRNA expressions and functional pathways linked to aging, providing a comprehensive view of the gene networks involved in the aging process (Kang et al. 2022).

### Proteomics in lactobacillomics approach

Proteomics could focus on the functional proteins involved in LAB species, especially metabolic pathways, stress responses, and probiotic properties, and may also discuss how proteomic analyses help identify biomarkers and adaptative mechanisms in LAB, which are crucial for optimizing health benefits and industrial applications in food and pharmaceuticals within the broader scope of Lactobacillomics. Proteomics may be the most promising area of Lactobacillomics since the number of studies is relatively fewer compared to genomics or metagenomics.

In LAB species, proteomics can provide insights into key metabolic pathways, including those involved in carbohydrate metabolism, fermentation processes, and the production of beneficial metabolites such as lactic acid, vitamins, and antimicrobial peptides. Understanding these pathways is crucial for optimizing probiotic efficacy and enhancing the production processes in food fermentation. LAB species are often exposed to various stressors, including changes in pH, temperature, and the presence of toxic compounds. Proteomic analyses enable researchers to identify proteins involved in stress response mechanisms, such as chaperones and antioxidant enzymes, which play significant roles in the survival and adaptation of these microorganisms under harsh conditions. This knowledge is essential for improving the resilience of probiotics during industrial applications.

One of the advanced techniques used in proteomic studies of LAB is the iTRAQ (Isobaric Tag for Relative and Absolute Quantitation) method. This isobaric labeling technique allows for the simultaneous quantification of proteins from different samples, providing a comprehensive view of protein expression levels. For instance, research has demonstrated the application of iTRAQ in studying *Lactiplantibacillus plantarum*, revealing its mechanisms for cadmium tolerance and the characterization of tolerance-related proteins, such as the prophage P2b protein 18 (Gu and Zhao 2019).

*Lactiplantibacillus plantarum*, a facultative heterofermentative lactic acid bacterium, is known for its robust adaptability to diverse environments, including the human gut and various fermented foods. This adaptability is attributed to its ability to modulate molecular mechanisms in response to fluctuating conditions, specifically through the regulation of a wide array of proteins. The regulatory network involves key repressors, HrcA and CtsR, and global regulators like carbon catabolite control protein A (CcpA). CcpA significantly manages responses to different oxygen levels, supporting *L. plantarum*’s metabolic flexibility. The proteomic analysis highlights that the absence of CcpA influences the transition between homolactic and mixed fermentation pathways. In anaerobic conditions, *L. plantarum* primarily produces lactic acid through homolactic fermentation. However, the shift to mixed fermentation under aerobic conditions enables the bacterium to generate additional byproducts, such as acetic acid, allowing for more efficient energy utilization (Mazzeo et al. 2012). The co-culture of *Limosilactobacillus reute*ri ZJ625 and *Ligilactobacillus salivarius* ZJ614 as a multi-strain probiotic preparation presents a promising approach to enhance probiotic efficacy. To understand the molecular basis of their interaction, this study employed liquid chromatography-mass spectrometry (LC-MS) proteomics to analyze both intracellular and extracellular proteomes during the mid-exponential growth phase. The analysis revealed differentially expressed proteins (DEPs) that shed light on the cooperative and adaptive responses between these strains (Kwoji et al. 2024).

The importance of *Lactobacillus acidophilus*’s proteomic adjustments to oxidative stress lies in its crucial response mechanisms, including cysteine biosynthesis, DNA repair, and energy metabolism. Oxidative stress is a common challenge in the industrial production of probiotics, as bacteria encounter reactive oxygen species (ROS) during various manufacturing processes. The proteomic adaptations of *Lactobacillus acidophilus* NCFM in response to oxidative conditions, using hydrogen peroxide (H₂O₂) as a stress inducer was investigated. By simulating an oxidative environment, researchers sought to identify protein abundance changes associated with survival and adaptation mechanisms. The investigation utilized 2D gel-based comparative proteomics, focusing on proteins with altered expression during the exponential growth phase. By leveraging these findings, probiotic manufacturers can optimize strains and processes to ensure product quality, stability, and efficacy in functional food applications. Further research could focus on enhancing specific pathways or exploring other stress factors, contributing to the advancement of probiotic science and technology (Calderini et al. 2017).

The S-layer, a lattice-like structure composed of self-assembling S-layer proteins (Slps), forms the outermost layer in many bacterial cells and plays a crucial role in various protective and functional processes. In *Levilactobacillus brevis*, a lactic acid bacterium commonly found in diverse environments like sourdough, dairy products, and the human gut, the S-layer is essential for probiotic activity. This layer not only shields the bacteria from environmental stresses but also aids in adhesion to host cells and modulates immune responses in the gut. Additionally, Slps serve as scaffolds for displaying other cell surface proteins known as S-layer-associated proteins (SLAPs), which further enhance bacterial interactions with the host and the environment.

The probiotic properties of *L. brevis*, including its role in inhibiting bacterial infections and boosting immune function, have been linked to the functions of Slps. In this study, researchers used a shotgun proteomic approach to analyze both Slps and SLAPs in five *L. brevis* strains isolated from traditional Southern Italian sourdoughs. The findings revealed unique surface protein patterns among these closely related strains, suggesting that variations in S-layer and associated proteins could influence the specific probiotic and biotechnological traits of each strain. This insight highlights the potential of harnessing *L. brevis* for tailored applications in probiotics and food biotechnology (Mazzeo et al. 2022).

### Metabolomics/metabonomics in lactobacillomics approach

Metabonomics/metabolomics is a science that involves metabolite changes, types, and quantities (Gu and Zhao 2019). Key tools in this science include liquid chromatography-mass spectrometry (LC-MS), gas chromatography-mass spectrometry (GC-MS), mass spectrometry (MS), and nuclear magnetic resonance (NMR) (Wang et al. 2023a). In the Lactobacillomics approach, metabolomics and metabonomics involve a comprehensive analysis of the metabolites produced by LAB species, which are key in probiotics and fermented foods (Liang et al. 2018; Gong et al. 2024; Fan et al. 2024). These analyses include; (i) profiling bioactive compounds such as assessing health-promoting metabolites such as short-chain fatty acids and vitamins to understand their contributions to gut health (Cheong et al. 2022; Ito et al. 2022; Yu et al. 2024), (ii) pathway analysis such as exploring nutrient utilization pathways by KEGG, and GABA synthesis to identify how these strains produce beneficial metabolites (Aguiar-pulido et al. 2016; Kwoji et al. 2023; Pan et al. 2023b), (iii) Strain-Specific Fingerprinting, highlighting unique metabolic profiles among different LAB strains, which can indicate specialized functions (Börner et al. 2019b; Gu and Zhao 2019), (iv) stress response such as examining how LAB species respond to environmental stressors, providing insights into their resilience in probiotic formulations (Aguiar-pulido et al. 2016), (v) dynamic changes such as tracking changes in metabolic profiles over time to understand adaptation in gut conditions, (vi) biomarker discovery, such as identifying metabolites as biomarkers for developing functional foods that target specific health outcomes (Qin et al. 2022). The metabolic activity of LAB during fermentation forms characteristic flavors. Some metabolites, such as organic acids, amino acids, oligosaccharides, and peptides, contribute to this characteristic. Metabonomics has been used to monitor this process (Liu et al. 2021). NMR and GC-MS have been used to evaluate the metabolic spectrum. The effect of calcium on EPS biosynthesis was researched with a metabolomics approach. Metabolomics analysis revealed significant changes in the small molecular metabolites in the tricarboxylic acid cycle, glucose metabolism, and propionic acid metabolism (Jiang et al. 2021). *Lacticaseibacillus casei* ATCC334 was used for jackfruit juice fermentation, and metabolomics changes were investigated (Muhialdin et al. 2021). LAB ability to metabolize tryptophan and generate various indole derivatives beneficial for human health was analyzed (Pan et al. 2023b).

### Advancing lactic acid bacteria research through multi-omics technologies

Multi-omics is the combination of single omics technologies mentioned above to increase a more comprehensive and integrative view of LAB. Multi-omics approach provides researchers to identify biomarkers, enhance predictive models, elucidate genetic potential, detect metabolic profiles, and characterize protein functions (Kwoji et al. 2023). Researchers used multi-omics to analyze LAB genomes, revealing that many LAB species possess genes for tryptophan breakdown, which are conserved across different strains, albeit with variations in gene sequence abundance. LAB was shown to produce multiple metabolites, such as indole-3-lactic acid (ILA), indole-3-acetic acid, and 3-indolealdehyde, though specific strains demonstrated unique metabolite production profiles (Pan et al. 2023b). As a new term flavoromics was applied to the fermentation process by a multi-omics approach. The unique flavor formation in oat beverages was investigated by co-fermented with LAB. Researchers integrated cross-correlation analysis and untargeted flavoromics approaches to reveal how LAB contributes to the aroma profile such as organic acids, free amino acids (FAAs), and volatile flavor compounds (VFCs) during fermentation (Yu et al. 2024). The critical role of cross protection in LAB under environmental stresses such as acidic, thermal, osmotic, and oxidative, which is essential for their resilience in food fermentation and production processes, has been extensively studied. However, the multi-omics approach is useful to detect stress response of LAB and underlying mechanism (Yang et al. 2021). Detection of genes, proteins, and metabolites provides valuable insight into their adaptive mechanisms. Through multi-omics analysis, researchers can identify key regulatory networks and protective mechanisms, such as chaperone proteins, osmoprotectants, and antioxidant systems, which enhance LAB resilience.

### Bibliometric analysis

**Fig. 1:**
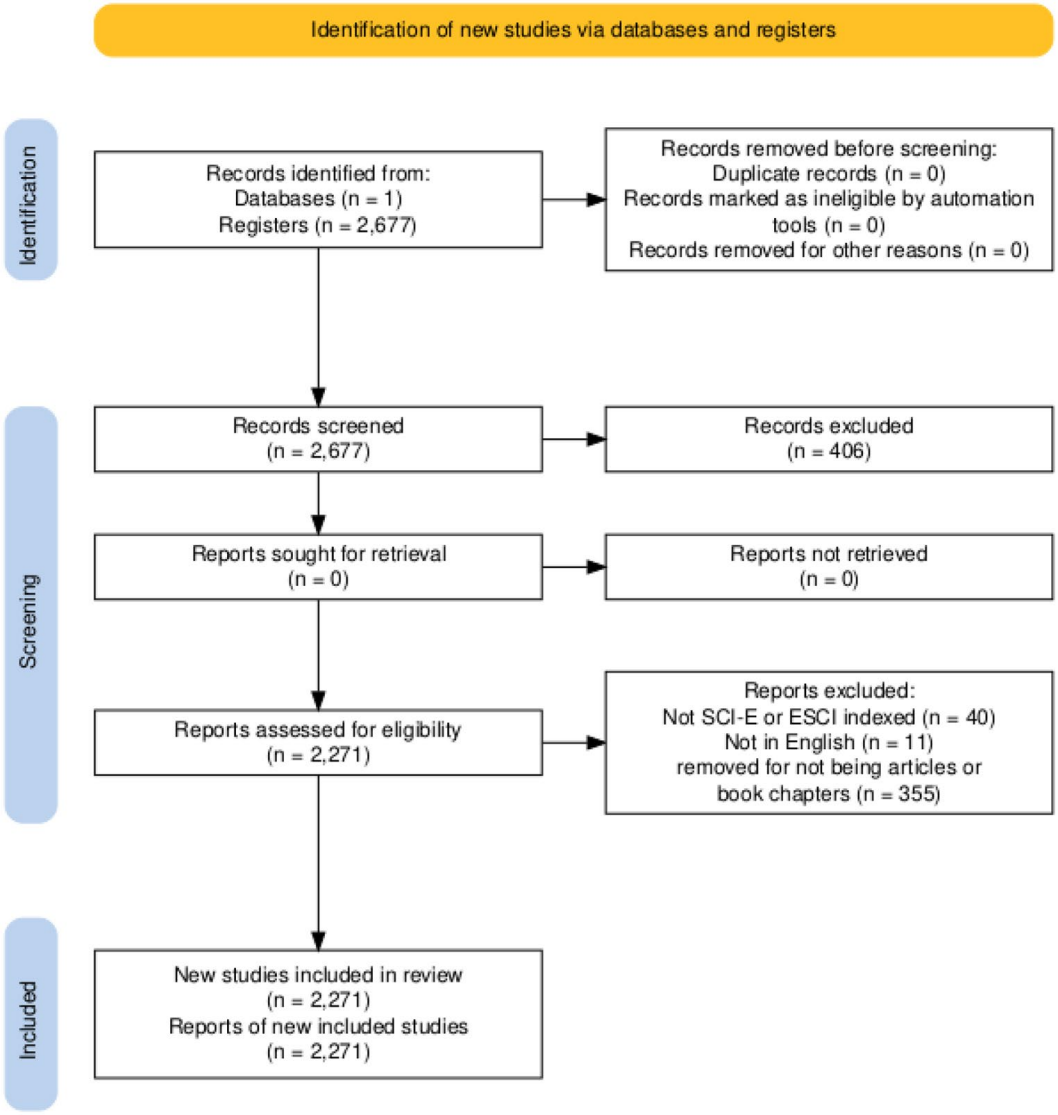
PRISMA flow diagram

Following the PRISMA diagram flow chart, 2677 documents were obtained in WoS using keywords. Only articles and book chapters were selected and this number decreased to 2322. SCI-E and ESCI were selected and the total number decreased to 2282, finally only publications in the English language were selected and the number decreased to 2271 (Fig. 1). The completeness of the metadata was excellent, good, or acceptable (keywords).

### Annual scientific and citation production

**Fig. 2:**
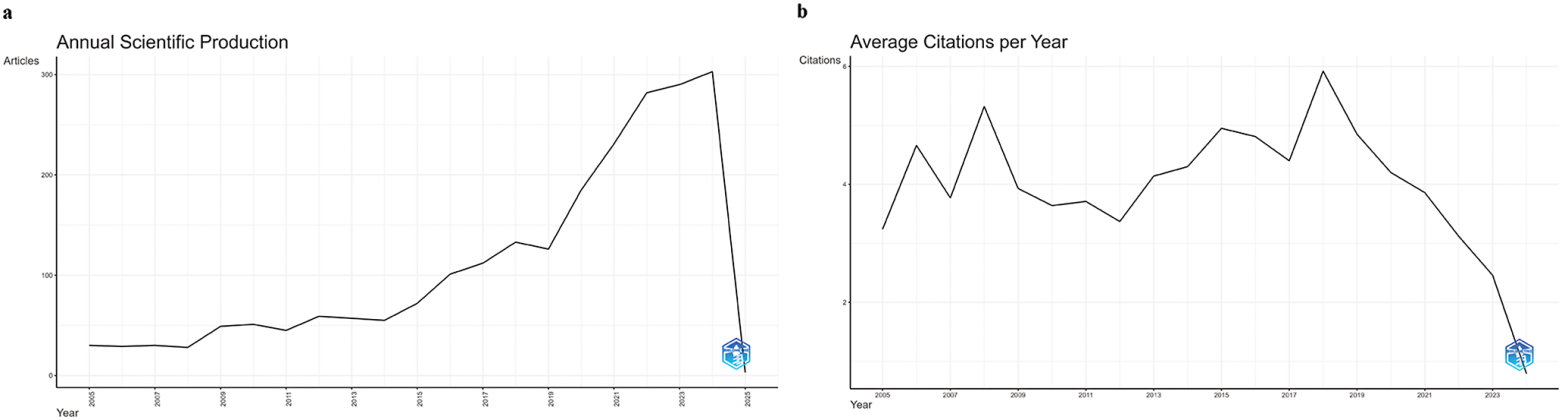
Annual Scientific Production (**a**) and Average Citation per Year (**b**)

Figure 2a implies a steady growth from 2006 to around 2016, the scientific production appears relatively low, with only minor fluctuations, suggesting that research interest in this area was modest during this period. However, a significant increase in publications is noticeable from around 2017, suggesting a growing interest in lactobacillomics and related topics. This may coincide with advancements in omics technologies and a growing understanding of the role of LAB in health and biotechnology. The sharp rise between 2020 and 2024, especially with a peak around 2023, highlights a considerable surge in research activities. This trend may reflect an increased focus on microbial health, probiotics, and multi-omics approaches in recent years.

Figure 2b implies that while research productivity has increased, as shown in the previous figures on scientific production, recent articles may not yet have had time to accumulate significant citations. Peaks in certain years are likely to correspond to seminal works or breakthroughs, while the decrease in average citations for recent years could simply be due to the citation lag effect. This aligns with typical patterns in academic publishing, where citation impact often becomes apparent only several years post-publication.

### Country collaboration

**Fig. 3:**
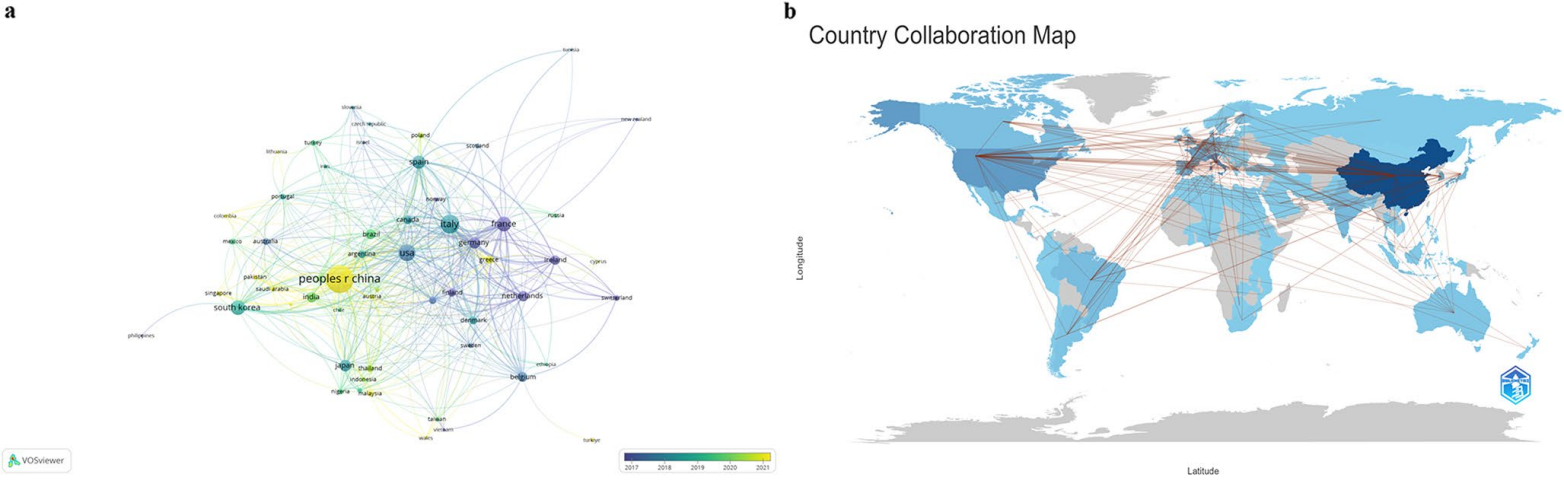
Country collaboration by year (**a**) and world collaboration map (**b**). Lines between countries indicate collaborative links, with more connections denoting stronger or more frequent research partnerships

Figure 3a represents countries that are connected based on co-authorship over time. The color gradient (from blue to yellow) shows the average year of research collaborations, so earlier collaborations are in blue, and more recent ones are in yellow. This visualization helps track the evolution of international research collaborations over time. Figure 3b shows the geographic distribution of collaborations globally. Countries are shaded in blue to indicate research activity levels, and connecting lines represent partnerships between countries. The USA, China, and several European countries such as Italy, France, and Spain appear to be central hubs, as they are connected to multiple countries. This suggests that these countries are significant contributors to international research collaborations in this field.

### Author’s keywords and research connections

**Fig. 4:**
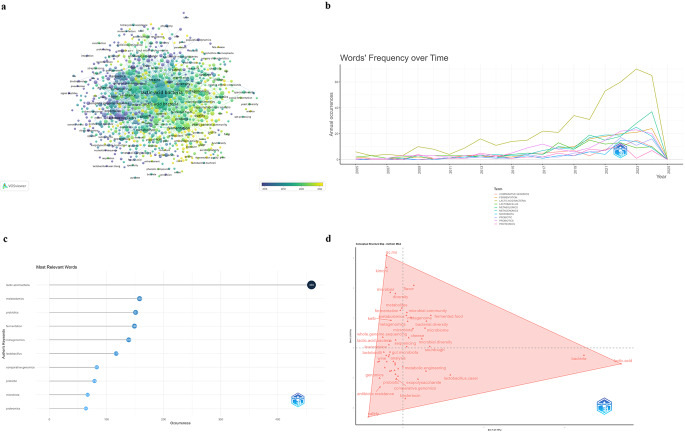
Key terms (**a**), word’s frequency over time (**b**), most relevant words (**c**), and Factorial Map (**d**)

Figure 4 represents key terms and research connections in the field of LAB (LAB) and probiotics through omics approaches. The largest nodes represent major themes in this field, with terms like “probiotics/probiotic”, “metagenomics”, “fermentation”, “antibiotic resistance”, and “diversity” appearing prominently. This suggests these are highly relevant topics in recent LAB research. The lines connecting the nodes indicate co-occurrence or collaboration between topics. For example, “probiotics” is closely connected with terms like “metagenomics,” “proteomics,” and “gut microbiota,” indicating a multidisciplinary approach to studying probiotics with advanced omics tools. The color coding from blue to yellow shows the timeline of publication frequency. Topics like “quality”, “safety” and “metabolites” appear in lighter colors, suggesting recent research interest (2022–2023). The map reflects the evolution of research interest from traditional probiotics and genomics toward more integrated omics techniques, such as proteomics and metabolomics, to study LAB comprehensively (Fig. 4a). Figure 4b shows the cumulative occurrences of key terms related to LAB research from 2005 to 2025. Each line represents the frequency of a specific term’s appearance over time, highlighting trends in research focus within this field. “Metagenomics” (dark blue line) shows the most significant growth, particularly after 2017, indicating increasing research interest in the genetic study of LAB and probiotics. “Lactic acid bacteria” (green-brown line) and “Probiotics” (purple line) also show a strong upward trend, reflecting a consistent and growing focus on probiotics. “Metabolomics” (dark green line) and “Metagenomics” (dark blue line), both lines show noticeable growth after 2015, suggesting that these omics techniques have become increasingly important in studying the metabolic profiles and microbial communities of LAB. Terms like “Proteomics” also gradually increase but remain less prominent than “Genomics”. Overall, the graph reflects a shift in research towards using advanced omics tools to study LAB, especially since 2015, aligning with the emergence of recent technologies in microbiome and genomic studies. The bar chart in Fig. 4c displays the most frequently occurring keywords in research related to LAB, reflecting topics with high relevance in recent studies. “Lactic Acid Bacteria” and “Metabolomics” keywords highlight a significant interest in microbial and metabolic aspects of LAB studies. “Metagenomics,” and “fermentation” appear as common terms, suggesting that various omics technologies are integral to understanding LAB on multiple levels (genomic, proteomic, and metabolic). Other keywords like “Proteomics”, “Fermentation,” and " Comparative Genomics” are also frequent but to a lesser extent, indicating specialized interests in comparative genomic approaches, fermentation processes, and general probiotic studies. This chart indicates a comprehensive approach to LAB research, where traditional topics like probiotics and LAB are increasingly complemented by advanced omics methodologies.

Figure 4d shows the factorial map (multiple correspondence analysis) of author’s keywords. All terms are grouped into a single cluster (Cluster 1), which suggests that the dataset represents keywords within a related research theme exploring interactions among omics fields (genomics, metabolomics, etc.) and lactobacilli-related topics. This single cluster reflects a tightly connected theme in the dataset, potentially showing the cohesive nature of research in this field where genomics, metabolism, and probiotic applications are intertwined.

Upper Region (Dim 2 Positive); this area includes terms like “microbial diversity,” “fermented food,” “metabolomics,” and “kimchi”. It likely represents studies on microbial ecosystems and food microbiology, particularly focused on diverse fermented foods. The presence of terms like “microbial community” and “bacterial diversity” suggests a strong interest in understanding how microbial diversity impacts fermentation processes and flavor profiles in foods such as kimchi, kefir, and cheese. Research in this area is typically concerned with analyzing microbiomes within fermented products and their effects on flavor, nutritional quality, and health. Lower Left Region (Dim 1 and Dim 2 Negative); this region contains terms like “genomics,” “antibiotic resistance,” “probiotic,” “safety,” and “comparative genomics”. This cluster may represent the field of probiotic genomics and safety assessment, focusing on genetic analysis and safety concerns related to LAB strains. Topics like “antibiotic resistance” are critical for assessing the safety of probiotic strains, especially given the health implications of antibiotic-resistant genes in food-related microbes. “Bacteriocin” and “exopolysaccharide” also appear here, suggesting research on bioactive compounds produced by *Lactobacillus*, which contribute to their probiotic and antimicrobial properties. Right Edge (Dim 1 Positive, Dim 2 Neutral); the terms “lactic acid” and “bacteria” appear more isolated on the right edge of the map, indicating they are frequently discussed in different contexts or general studies on LAB. This region likely represents fundamental research focusing on lactic acid production, a defining characteristic of *Lactobacillus* species, and their use in various industrial applications. The presence of “metagenomics,” “multi-omics,” and “whole genome sequencing” indicates that researchers are increasingly using comprehensive omics approaches to study LAB in complex microbial communities. These methods provide insights into how LAB interacts with other microbes and contributes to the microbiome within fermented foods and the human gut, paving the way for precision nutrition and personalized probiotics. Terms such as “cheese,” “wine,” “fermentation,” and “sourdough” suggest a focus on using LAB in traditional food fermentations. This cluster indicates studies centered on how LAB strains contribute to specific textures, flavors, and preservation methods in these foods. “Metabolic engineering” appears within this group, highlighting efforts to optimize LAB strains for enhanced performance in food fermentation, such as improving flavor profiles, shelf-life, or nutrient content. The appearance of terms like “gut microbiota,” “microbiome,” “probiotic,” and “safety” underlines research on the health benefits and safety of LAB as probiotics. Functional studies often involve characterizing the interactions between LAB strains and host microbiomes, exploring their potential to enhance gut health, immune response, and metabolic processes. Terms like “sequencing,” “analysis,” and “comparative genomics” imply an emphasis on high-throughput sequencing and bioinformatic analysis within lactobacillomics research. The term “gc.ms” (gas chromatography-mass spectrometry) indicates the use of advanced analytical techniques to investigate the metabolic byproducts of LAB, often in the context of food fermentation and flavor development.

The presence of “metataxonomics” and “metabonomics” suggests that niche areas within omics research are gaining traction in LAB studies. These emerging fields focus on identifying and categorizing microbial communities and their metabolic outputs, offering valuable insights into complex microbial ecosystems like those found in fermented foods and the gut.

### The most cited and relevant sources

**Fig. 5:**
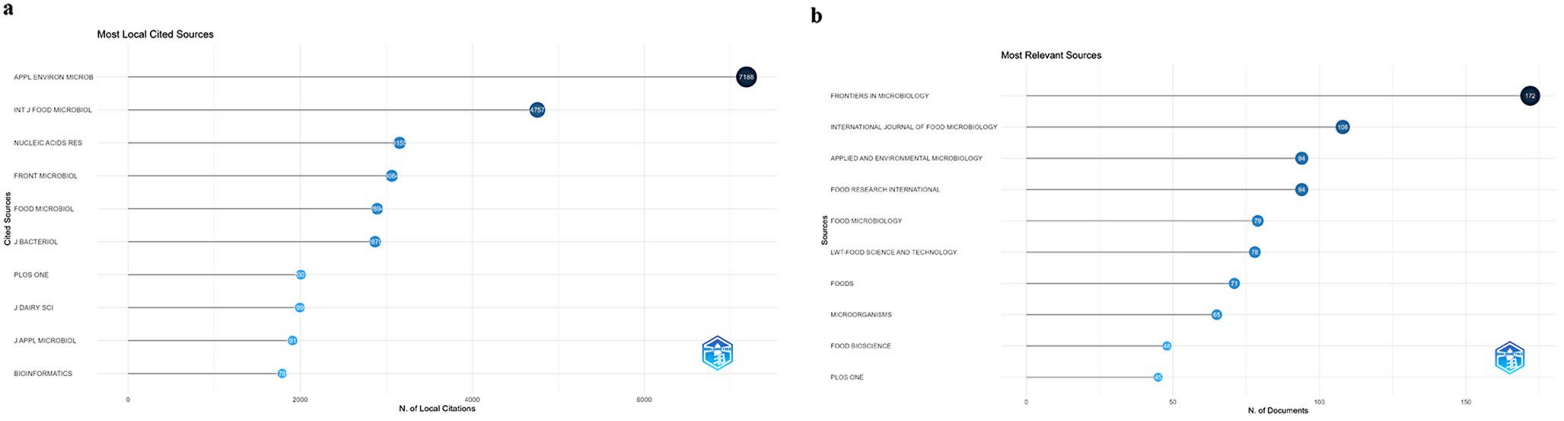
Most local cited (**a**) and most relevant sources (**b**)

Figure 5a and b represent the journals that are highly cited, widely read, or published in leading journals in the field. Applied and Environmental Microbiology was the most locally cited source while Frontiers in Microbiology was the most relevant source for the Lactobacillomics approach.

This citation and source relevance analysis highlights the interdisciplinary nature of Lactobacillomics, bridging microbiology, genomics, food science, and health. These journals are instrumental in advancing the understanding of LAB’s roles in probiotics, food safety, and microbiome science.

### Thematic map and trend topics

**Fig. 6:**
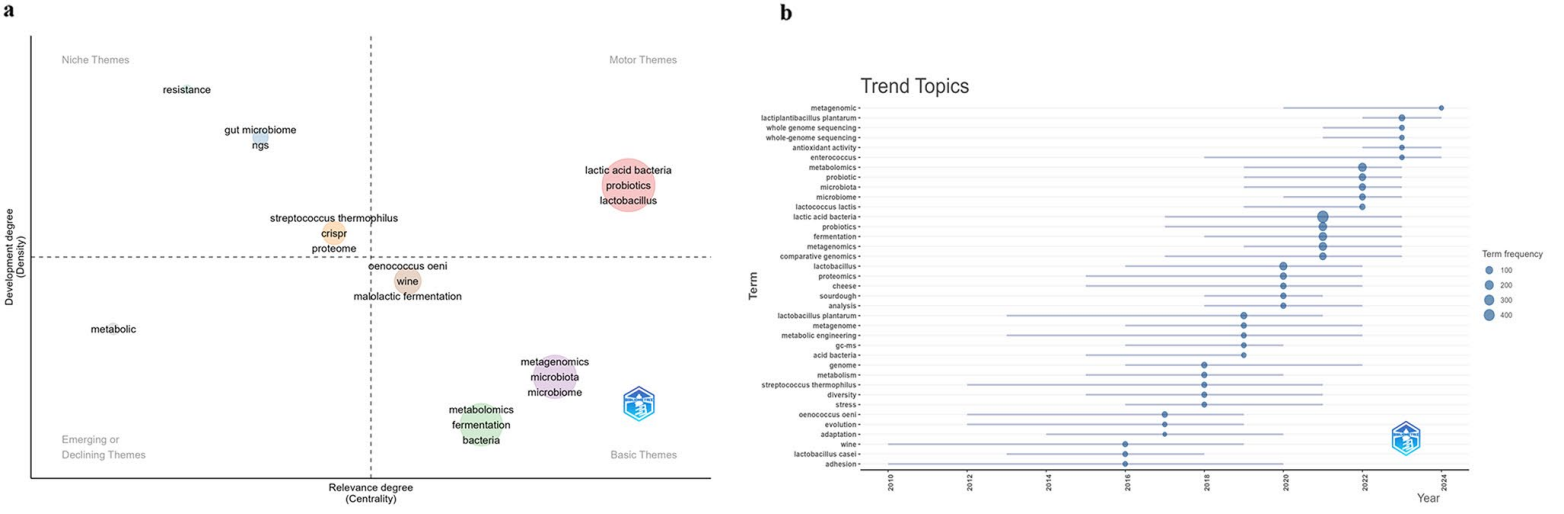
Thematic map (**a**) and Trend Topics (**b**)

Thematic map categorizes key themes in LAB research based on Development Degree (Density) and Relevance Degree (Centrality) across four main quadrants (Fig. 6a).

Located in the upper right quadrant, these are well-developed and central topics in lactobacillomics, representing essential and actively researched themes. The focus here is on LAB species, their probiotic functions, and the production of lactic acid, which are fundamental to the field. These themes are likely driving much of the research interest due to their relevance to health and industry, emphasizing both the functional benefits and applications of LAB in probiotic products.

Niche Themes (High Density, Low Centrality); “Resistance, gut microbiome, NGS”, this quadrant holds specialized but less central themes, such as antibiotic resistance, gut microbiome studies, and next-generation sequencing (NGS) technologies. These topics may be highly specific and technically complex, focusing on advanced genetic analyses and the study of resistance genes within the LAB microbiome. They are significant for understanding the genomic and ecological roles of LAB but may not be widely connected to other research themes.

Basic Themes (High Centrality, Low Density); “Metabolomics, fermentation, bacteria” and “Metagenomics, microbiota, microbiome”, found in the lower right quadrant, these themes are foundational but underdeveloped, suggesting they are important across multiple studies but might require further research to build depth. Metabolomics and fermentation are core topics in the study of LAB due to the focus on metabolic outputs in fermented foods. Similarly, metagenomics and microbiota research are crucial for understanding microbial ecosystems involving LAB and other bacteria. These themes form a base of essential knowledge, providing context and background for more specialized studies.

Emerging or Declining Themes (Low Centrality, Low Density), “Metabolic”, this term is found in the lower left quadrant, which includes either emerging areas with potential growth or declining topics with decreasing relevance. The term metabolic alone may reflect early-stage exploration into metabolic pathways and activities specific to LAB. The sparse positioning here could indicate a new area of interest or one that’s becoming less emphasized in recent research.

Terms such as “*Streptococcus thermophilus*,” “CRISPR,” “proteome,” “*Oenococcus oeni*” “malolactic fermentation,” and “wine” occupy central yet low-density areas, suggesting these are specialized topics that intersect with broader lactobacillomics themes.

The presence of *Streptococcus thermophilus* and CRISPR hints at genetic editing tools and the study of specific bacteria that may complement *Lactobacillus* in various applications, such as dairy fermentations.

*Oenococcus oeni* and malolactic fermentation are crucial in the context of wine production, highlighting the role of other LAB in industries beyond general food fermentation.

The trend topic given in Fig. 6b presents research trends over time-related to Lactobacillomics. The most recent terms with high frequency, indicated by larger dots toward 2024, include “metagenomics,” “probiotics,” “microbiome,” and “antioxidant activity.” These fields have gained attention in recent years. Some terms have been researched continuously over the years. For example, “lactic acid bacteria,” “probiotics,” and “metagenome” show consistent interest across the timeline. This indicates metagenome notion has evolved previously while the metagenomics notion was relatively new. Terms such as “metagenomics,” “whole genome sequencing,” “proteomics,” and “metabolomics” have increased in frequency, reflecting a trend toward using omics technologies for a deeper understanding of microbial functions and interactions.

**Fig. 7:**
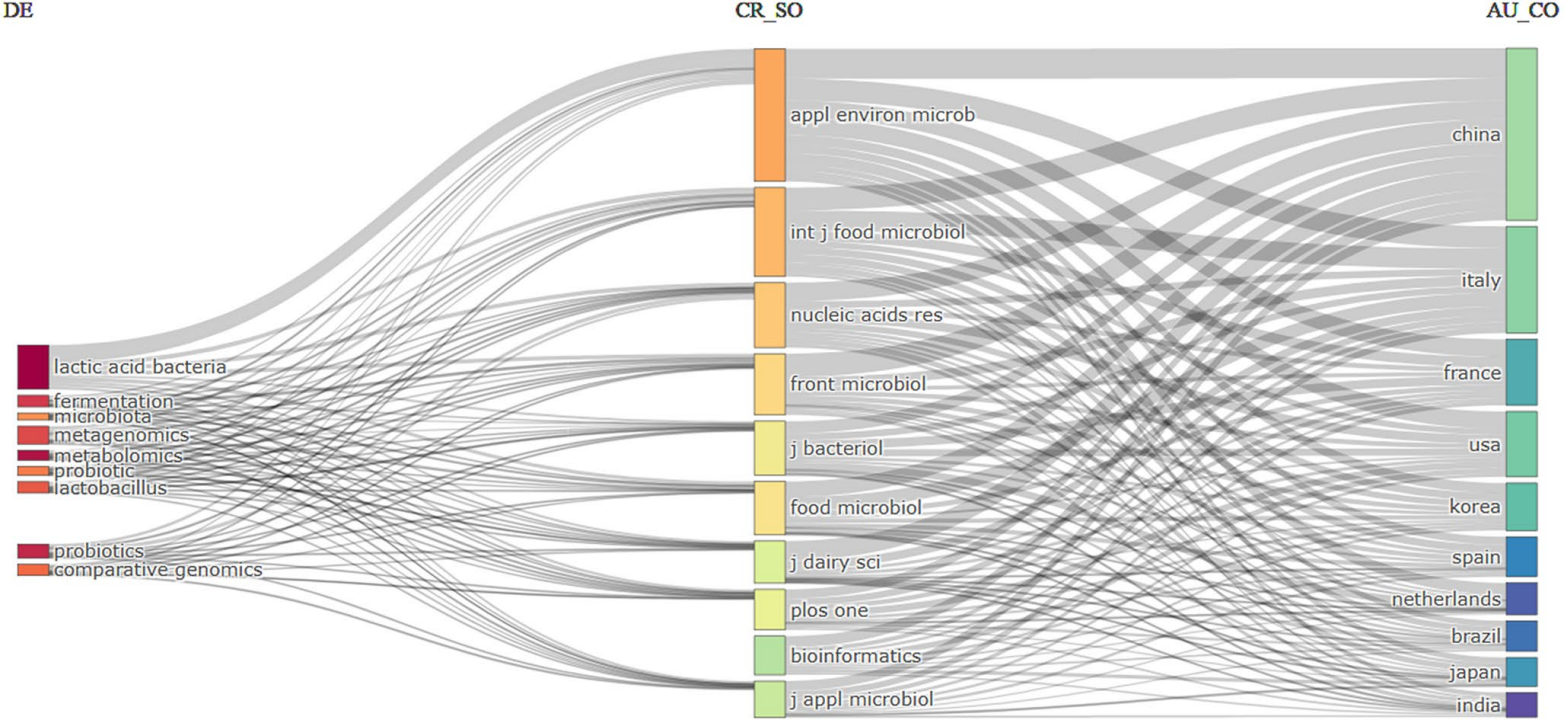
Three-Field Plot (Sankey Diagram). AU_CO: Author’s country, SO: Soources, DE: Author’s keywords or Research Topics

Figure 7 highlights the interdisciplinary nature of LAB research and its global collaboration network across prominent journals and research institutions in various countries. Major topics include “lactic acid bacteria,” “fermentation,” “microbiota,” “metagenomics,” “metabolomics,” “probiotic,” and “*lactobacillus*.” These topics are widely studied and appear as primary keywords in publications. The primary contributing countries are China, Italy, France, the USA, and Korea, indicating strong global interest, particularly from Asia, Europe, and North America. Key journals publishing research in this field include *Applied and Environmental Microbiology* (appl environ microb), *International Journal of Food Microbiology* (int j food microbiol), Nucleic Acids Research (nucleic acids-res), *Frontiers in Microbiology* (front microbiol), *Journal of Bacteriology* (j bacteriol). LAB, fermentation, and probiotics are closely linked to journals such as *Applied and Environmental Microbiology* and *Frontiers in Microbiology*, which are published widely on these topics. Countries like China and Italy show connections to multiple topics and journals, suggesting active research communities with a broad focus within this domain.

## Conclusion

Overall, bibliometric insights suggest that LAB research is moving towards a more holistic, interdisciplinary approach, leveraging advanced omics tools to understand microbial functions and interactions. This field holds promising applications across health, food, and biotechnology, setting the stage for breakthroughs in personalized nutrition, microbiome therapies, and sustainable food systems. Therefore, as a new term, Lactobacillomics provides a focused framework for studying the genus *Lactobacillus* through a comprehensive, multi-omics approach. By integrating genomics, proteomics, metabolomics, and other omics technologies, Lactobacillomics enables researchers to gain detailed insights into the metabolic pathways, genetic diversity, and functional roles of *Lactobacillus* species in various environments, including the human gut, fermented foods, and ecological niches. In summary, Lactobacillomics represents a specialized avenue within LAB research, enabling precision science and tailored applications that harness the unique properties of *Lactobacillus* for health and industry. From the early 2010s to 2023, the trend shows progress from foundational studies on LAB properties to advanced “-omics” research aiming to understand LAB in complex systems and their potential applications in health and food industries. The image highlights how the field has evolved with technological advances and a growing focus on integrating LAB into health-promoting and sustainable food systems.

## Data Availability

No datasets were generated or analysed during the current study.
